# Chemotherapy Improved Prognosis of Mesenchymal Chondrosarcoma with Rare Metastasis to the Pancreas

**DOI:** 10.1155/2014/249757

**Published:** 2014-01-30

**Authors:** Shinji Tsukamoto, Kanya Honoki, Akira Kido, Hiromasa Fujii, Yasunori Enomoto, Chiho Ohbayashi, Yasuhito Tanaka

**Affiliations:** ^1^Department of Orthopaedic Surgery, Nara Medical University, 840 Shijo-cho, Kashihara, Nara 634-8521, Japan; ^2^Department of Diagnostic Pathology, Nara Medical University, 840 Shijo-cho, Kashihara, Nara 634-8521, Japan

## Abstract

Extraskeletal mesenchymal chondrosarcoma is rare and metastasis to the pancreas is extremely rare, with only four cases reported in the literature. The therapeutic effectiveness of chemotherapy remains uncertain. We report a 39-year-old man with extraskeletal mesenchymal chondrosarcoma of the buttock, who had metastases to the pancreas, bones, and lung. He underwent distal pancreatectomy, resection of the buttock tumor, and chemotherapy. He had a good response to chemotherapy and survived for about 3 years after surgery.

## 1. Introduction

Mesenchymal chondrosarcoma is a rare malignant neoplasm characterized by a biomorphic pattern of poorly differentiated small round cells and islands of well-differentiated hyaline cartilage. This tumor has a widespread distribution. The craniofacial bones (especially the jaw bones), ribs, ilium, and vertebrae are the most common sites. One-fifth to one-third of the lesions are extraskeletal, primarily affecting the somatic soft tissues [1].

Metastasis to the pancreas is extremely rare, with only four cases reported in the literature [2–6]. We herein report a 39-year-old man with extraskeletal mesenchymal chondrosarcoma of the buttock, with metastasis to the pancreas.

## 2. A Case Report

**Figure 1:**
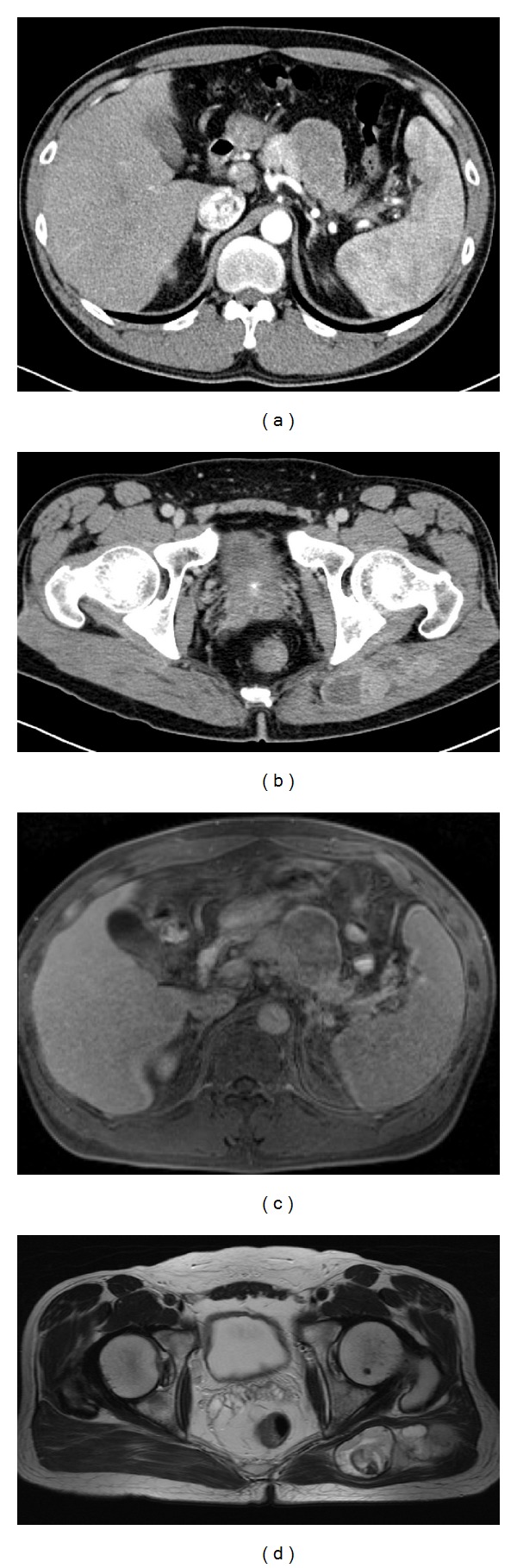
(a) Abdominal computed tomography, showing a well-circumscribed, low-attenuation, inhomogeneously enhancing mass measuring about 5 cm in diameter in the body and tail of the pancreas. (b) Abdominal computed tomography, showing a well-circumscribed, heterogeneously enhancing mass measuring about 8 cm × 3 cm in the left buttock. (c) Abdominal magnetic resonance imaging, showing a well-circumscribed mass measuring 4.5 cm in diameter in the body and tail of the pancreas. The mass had inhomogeneous enhancement with gadolinium diethylenetriaminepentaacetic acid on T1-weighted fat-suppression images. (d) Magnetic resonance imaging of the lesion in the left buttock, showing an inhomogeneously hyperintense mass on T2-weighted images.

A 39-year-old man presented with a 4-month history of severe upper abdominal pain after drinking alcohol. The patient denied any significant past medical or surgical history. Physical examination did not reveal any abdominal mass, and laboratory data including tumor markers (CEA, CA19-9, and DUPAN-2) showed no abnormalities. Abdominal computed tomography (CT) showed a well-circumscribed, low-attenuation, inhomogeneously enhancing mass measuring about 5 cm in diameter in the body and tail of the pancreas. There were no signs of invasion of the surrounding tissues or dilatation of the distal pancreatic ducts. The CT findings were not typical for pancreatic cancer (Figure 1(a)). CT also showed a well-circumscribed, heterogeneously enhancing mass measuring about 8 cm × 3 cm in the left buttock (Figure 1(b)). Magnetic resonance imaging of the abdomen showed a well-circumscribed mass measuring 4.5 cm in diameter in the body and tail of the pancreas. The mass was hypointense with punctate hyperintense areas on T1-weighted images and slightly hyperintense on T2-weighted images and showed inhomogeneous enhancement with gadolinium diethylenetriaminepentaacetic acid on T1-weighted fat-suppression images (Figure 1(c)). The radiological differential diagnosis included neoplasm of the endocrine pancreas and solid pseudopapillary tumor. Magnetic resonance imaging of the left buttock showed a tumor mass that was inhomogeneously hypointense on T1-weighted images and inhomogeneously hyperintense on T2-weighted images (Figure 1(d)). Abnormal lesions suspicious for metastases were observed in the sacral bones, ilium, and ischium. Chest CT showed a well-circumscribed mass measuring about 5 mm in diameter in the lower lobe of the right lung (S8), which was suspicious for metastasis. The patient underwent endoscopic ultrasonography-guided fine needle aspiration biopsy of the pancreatic lesion and needle biopsy of the buttock lesion.

**Figure 2:**
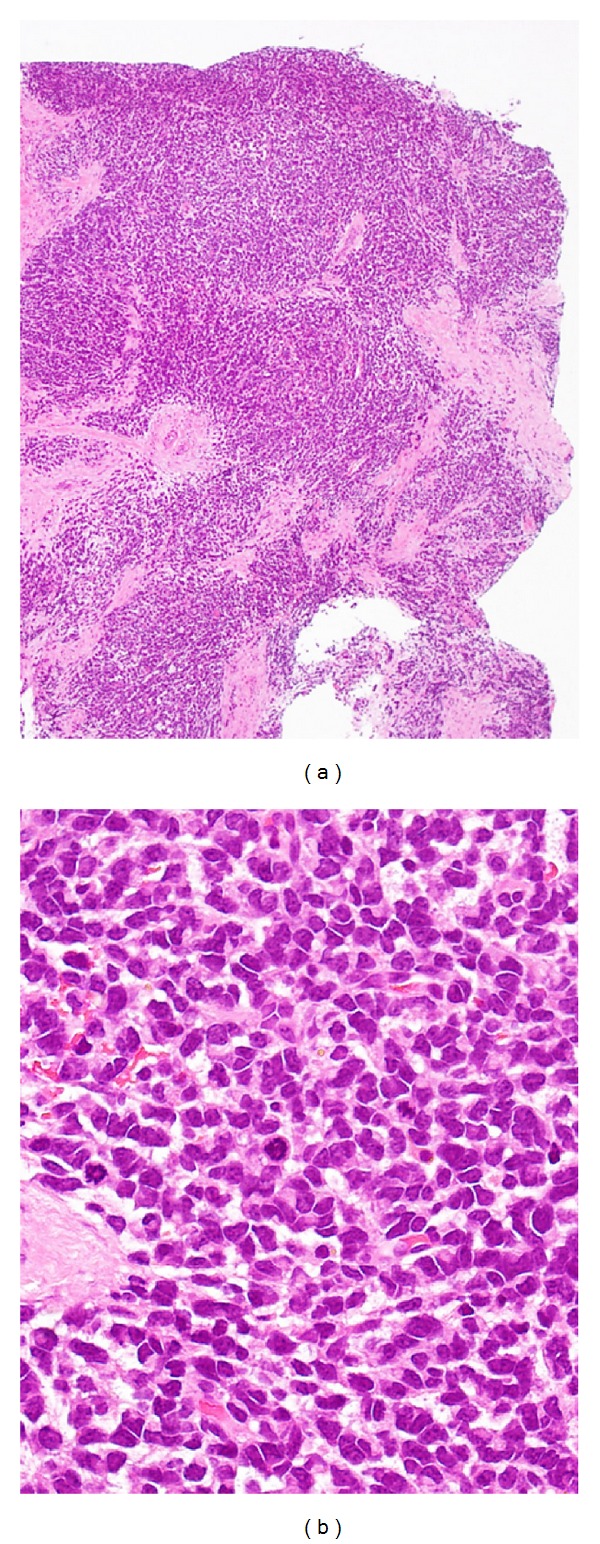
Histological findings of the biopsy specimen of the buttock tumor, showing a poorly differentiated round cell malignant neoplasm, growing in sheets. The tumor cells had scant, pale eosinophilic cytoplasm. Hematoxylin and eosin staining, original magnification ×100 (a) and ×400 (b).

Histological examination of both specimens showed a poorly differentiated round cell malignant neoplasm, growing in sheets. The tumor cells had scant, pale eosinophilic cytoplasm (Figure 2). These findings indicated a malignant tumor, but it was difficult to obtain a definitive histological diagnosis. We therefore performed distal pancreatectomy and wide resection of the buttock tumor to obtain a definitive diagnosis and determine the therapeutic strategy, including the chemotherapy regimen.

**Figure 3:**
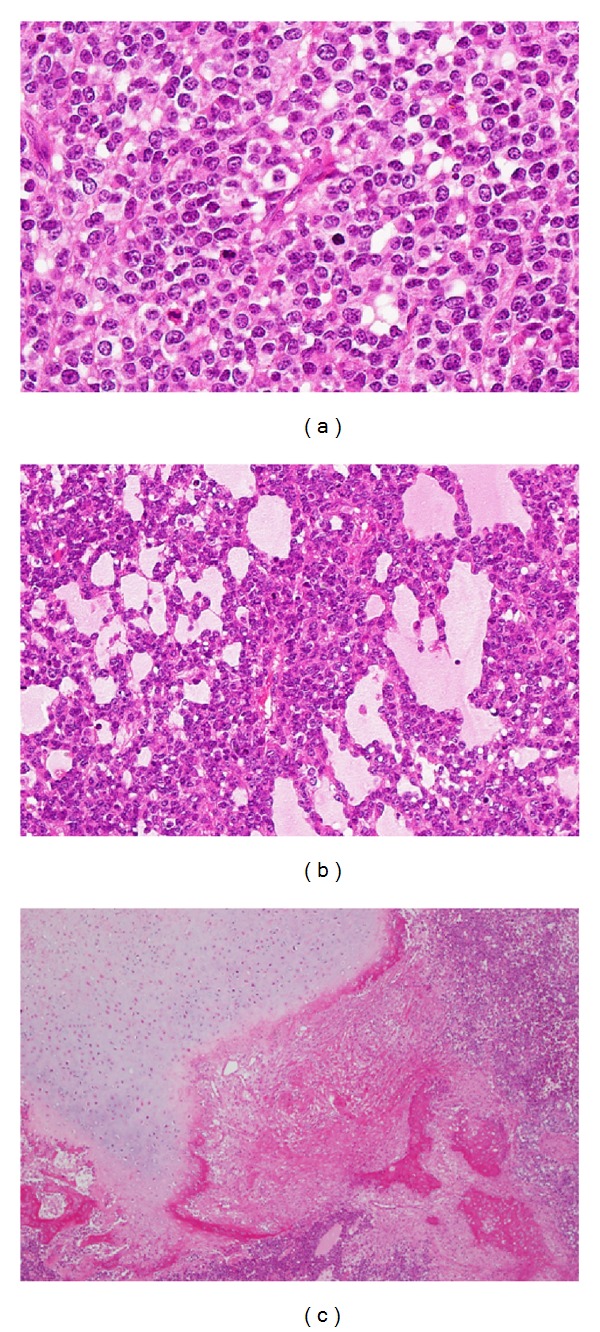
Histological examination of the resected buttock tumor, showing a poorly differentiated round cell malignant neoplasm, growing in sheets. The tumor cells had scant, pale eosinophilic cytoplasm. Hematoxylin and eosin staining, original magnification ×400 (a) and ×200 (b). There were large areas of well-differentiated cartilage, showing focal endochondral ossification ((c), ×40).

Intraoperative examination showed that the intra-abdominal tumor occupied the body and tail of the pancreas and pushed against the lesser curvature of the stomach. No ascites, peritoneal dissemination, or infiltration into the surrounding tissues was observed, and the tumor was resected. The resected tumor mass measured 6 cm × 5 cm. The buttock tumor had poorly defined margins with invasion into the gluteus maximus muscle and was widely resected with the muscle. The resected tumor mass measured 7.2 cm × 5.6 cm. Histological examination of both specimens showed a poorly differentiated round cell malignant neoplasm growing in sheets, with extensive necrosis. The tumor cells had scant, pale eosinophilic cytoplasm (Figures 3(a) and 3(b)). The buttock tumor had large areas of well-differentiated cartilage, with focal areas of endochondral ossification (Figure 3(c)). Immunostaining for pan-cytokeratin, chromogranin, synaptophysin, and desmin was negative, but there was diffuse membranous immunopositivity for CD99 in the vast majority of tumor cells. Fluorescence in situ hybridization revealed no evidence of *EWSR1* gene rearrangement. These histological and genetic findings led to a diagnosis of extraskeletal mesenchymal chondrosarcoma. As the tumor in the buttock was larger and was more typical of extraskeletal mesenchymal chondrosarcoma based on the histological findings, we concluded that the primary site was in the buttock and that the pancreatic tumor was metastatic.

**Figure 4:**
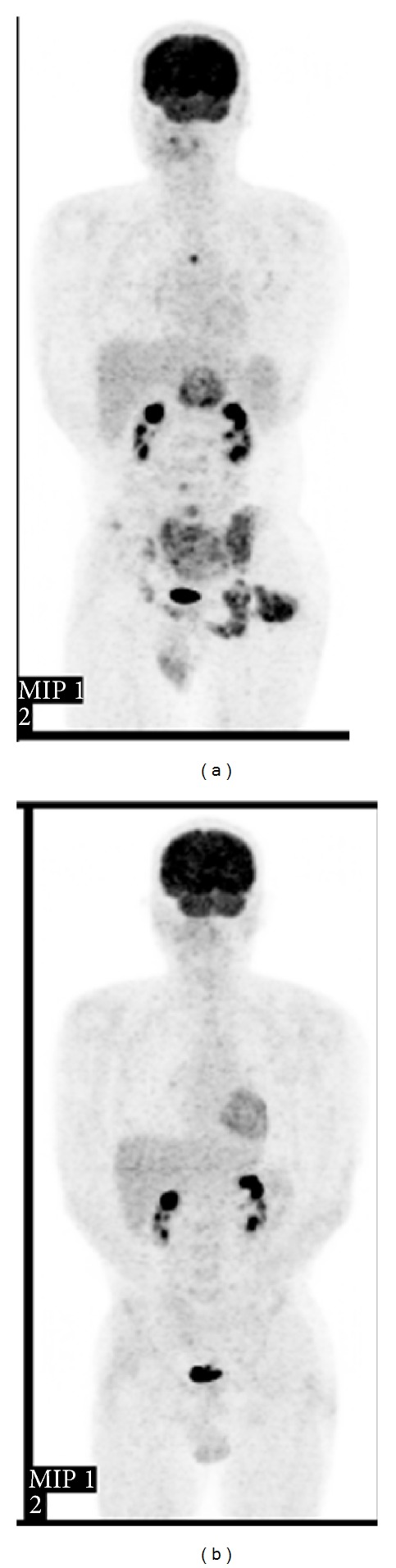
(a) Preoperative positron emission tomography, showing areas of increased uptake in the pancreas, left buttock, pelvis, and the thoracic, lumbar, and sacral vertebrae. (b) The areas of increased uptake disappeared after eight cycles of chemotherapy.

The patient received postoperative adjuvant chemotherapy consisting of three cycles of ifosfamide and doxorubicin, three cycles of cisplatin and doxorubicin, and two cycles of vincristine, doxorubicin, and cyclophosphamide to treat his multiple bone and lung metastases. He had a good response to chemotherapy, and most of the metastases were no longer detectable on positron emission tomography at the end of therapy (Figure 4). However, the metastatic tumors relapsed at 1 year and 3 months after surgery. The patient received additional chemotherapy consisting of one cycle of ifosfamide and doxorubicin, one cycle of ifosfamide and etoposide, one cycle of vincristine, doxorubicin, and cyclophosphamide, and four cycles of gemcitabine and docetaxel. The additional chemotherapy failed to suppress the tumor growth, and he died at 2 years and 10 months after surgery.

## 3. Discussion

Mesenchymal chondrosarcoma was first reported by Lichtenstein and Bernstein in 1959 [6], and extraskeletal mesenchymal chondrosarcoma in the soft tissues was reported by Dowling in 1964 [7]. The incidence of extraskeletal mesenchymal chondrosarcoma is very low. Shapeero et al. [8] reported that only 7 out of 224 cases of mesenchymal chondrosarcoma were extraskeletal in origin. The disease typically presents between the ages of 15 and 35 years and is more frequent in females than in males [9].

Mesenchymal chondrosarcoma is an aggressive malignancy, and the primary treatment is surgical resection. Huvos et al. [10] published a retrospective series of nine patients with mesenchymal chondrosarcoma treated with preoperative chemotherapy, and reported that three patients had a complete response, three had a partial response (all T-10 or T-11 protocols), and three did not respond (all high-dose methotrexate monotherapy protocols). Nooij et al. [11] reported a good pathological response of the primary tumor in one out of two patients who received preoperative doxorubicin-cisplatin combination therapy but only a limited effect in four patients with metastatic disease treated with the same regimen (two with stable disease). Cesari et al. [12] studied 21 mesenchymal chondrosarcoma patients who achieved complete surgical remission and reported a disease-free survival rate of 76% in those who received chemotherapy (9 patients) and 17% in those who did not receive chemotherapy (12 patients) (*P* = 0.008), suggesting that addition of chemotherapy improves disease-free survival. Several cases that were sensitive to combined radiotherapy and chemotherapy have also been reported [13, 14]. Tumors with a high percentage of small cells and limited cartilage content are thought to be the most sensitive to chemotherapy and radiotherapy [10], as with other small cell sarcomas. Although prospective studies are lacking, mesenchymal chondrosarcomas are considered to be sensitive to doxorubicin-based combination chemotherapy, as used in other bone tumors. Patients with mesenchymal chondrosarcoma should therefore be considered for adjuvant chemotherapy or for palliative chemotherapy if they have advanced disease [15].

The prognosis of patients with mesenchymal chondrosarcoma is poor because of the high frequencies of early recurrence and remote metastasis [8, 15]. The reported survival rates at 5 and 10 years are 54.6% and 27.3%, respectively [9].

Mesenchymal chondrosarcoma with metastasis to the pancreas is extremely rare, with only four cases reported in the literature. In this case, the patient presented with both a pancreatic tumor and a soft tissue tumor in the buttock, and it was difficult to determine the definitive diagnosis by biopsy. Because the patient was young and complained of abdominal pain, we resected both tumors to determine the definitive diagnosis and determine the therapeutic strategy, including chemotherapy. Although the prognosis was expected to be poor because of the widespread metastases to the pancreas, bones, and right lung, chemotherapy was beneficial and the patient survived for 2 years and 10 months after surgery.

We report here a rare case of mesenchymal chondrosarcoma with metastasis to the pancreas. Further development of novel agents for treatment is essential for improving the prognosis of this type of tumor.
